# Formulation Optimization and *In Vivo* Proof-of-Concept Study of Thermosensitive Liposomes Balanced by Phospholipid, Elastin-Like Polypeptide, and Cholesterol

**DOI:** 10.1371/journal.pone.0103116

**Published:** 2014-07-28

**Authors:** Sun Min Park, Jae Min Cha, Jungyong Nam, Min Sang Kim, Sang-Jun Park, Eun Sung Park, Hwankyu Lee, Hyun Ryoung Kim

**Affiliations:** 1 Drug Delivery System Group, Bio Research Center, Samsung Advanced Institute of Technology (SAIT), Yongin, Gyeonggi-do, South Korea; 2 Department of Chemical Engineering, Dankook University, Yongin, Gyeonggi-do, South Korea; Case Western Reserve University, United States of America

## Abstract

One application of nanotechnology in medicine that is presently being developed involves a drug delivery system (DDS) employing nanoparticles to deliver drugs to diseased sites in the body avoiding damage of healthy tissue. Recently, the mild hyperthermia-triggered drug delivery combined with anticancer agent-loaded thermosensitive liposomes was widely investigated. In this study, thermosensitive liposomes (TSLs), composed of 1,2-dipalmitoyl-*sn*-glycero-3-phosphocholine (DPPC), 1,2-distearoyl-*sn*-glycero-3-phosphoethanolamine-*N*-[methoxy(polyethyleneglycol)-2000] (DSPE-PEG), cholesterol, and a fatty acid conjugated elastin-like polypeptide (ELP), were developed and optimized for triggered drug release, controlled by external heat stimuli. We introduced modified ELP, tunable for various biomedical purposes, to our thermosensitive liposome (e-TSL) to convey a high thermoresponsive property. We modulated thermosensitivity and stability by varying the ratios of e-TSL components, such as phospholipid, ELP, and cholesterol. Experimental data obtained in this study corresponded to results from a simulation study that demonstrated, through the calculation of the lateral diffusion coefficient, increased permeation of the lipid bilayer with higher ELP concentrations, and decreased permeation in the presence of cholesterol. Finally, we identified effective drug accumulation in tumor tissues and antitumor efficacy with our optimized e-TSL, while adjusting lag-times for systemic accumulation.

## Introduction

A drug delivery system (DDS), developed for the highest efficacy of clinical drugs, provides damaged/diseased sites in a body with a therapeutic dosage of drugs, while reducing side effects caused by undesired delivery to normal tissues. The development of nanotechnologies has enabled specific tailoring of the properties of drug delivery carriers for currently existing medical issues and problems. Accordingly, the range of DDS applications in biomedical and clinical fields has expanded with the creation of unprecedented therapeutic approaches in the area of nanomedicine [1]. A platform technology of DDS has been adopted to potentiate a variety of diagnostic methodologies using biomarkers/bioimaging, tissue engineering/regenerative medicine, and gene therapy, as well as pharmaceutical industries [2]–[4].

Advances in nanotechnology have allowed for the fabrication of nano-sized particles that are capable of carrying a variety of drugs [5]. Selective drug targeting through the use of drug-loaded nanocarriers allows nanoparticles to effectively extravasate and accumulate in tumorous tissues exhibiting the enhanced permeability and retention (EPR) effect, constituted by leaky vasculature and poor lymphatic drainage [6]. Local mild hyperthermia, which conditions the treated site at about 42°C (39–42°C), has been applied clinically, in order to increase vascular permeability and interstitial microconvection of the drug within the sites of disease. This enhances the EPR effect, further elevating drug accumulation. Moreover, many researchers have developed thermosensitive nanovehicles to control drug release within the diseased region under locally-treated mild hyperthermia [6]–[13]. This could yield a synergistic effect with EPR, and provide abrupt exposure of highly concentrated drugs specifically to diseased tissues, further maximizing the therapeutic effects. Approved for clinical use, liposomes are currently one of the most versatile nano-sized drug carriers; thus, thermosensitive liposome (TSL) has been widely studied for transitioning laboratory findings to the clinic [1], [7], [9], [14].

Herein, we report the development of novel temperature-sensitive liposomes, a short chain elastin-like polypeptide-incorporating TSL (e-TSL), which is composed of 1,2-dipalmitoyl-*sn*-glycero-3-phosphocholine (DPPC), 1,2-distearoyl-*sn*-glycero-3-phosphoethanolamine-*N*-[methoxy(polyethyleneglycol)-2000] (DSPE-PEG), cholesterol, and a fatty acid conjugated elastin-like polypeptide (ELP). By incorporating ELP, consisting of a [VPGVG]n pentapeptide repeat, e-TSLs could possess high thermosensitivity and biocompatibility for use in various biomedical applications [15]–[18]. In this study, the component optimization of e-TSL was conducted as a thermosensitive drug carrier under mild hyperthermia. To select ELP-lipid conjugates for incorporation into the thermosensitive liposome bilayer, their transition temperature were obtained at different concentrations. To optimize the composition, the cargo-release from various liposomes, consisting of different molar ratios of main lipid (DPPC and DSPC), cholesterol, and selected ELP, was investigated. A simulation study supported the experimental data, which demonstrated the change of lateral diffusion coefficients with different ratios of ELP and cholesterol. Finally, the e-TSLs formulated with the optimized components were loaded with doxorubicin, and applied to tumor-bearing mice via intravenous injection, in order to obtain the largest amount of drug accumulation and better antitumor effect.

## Materials and Methods

### Ethics Statement

All animal protocols were reviewed and approved by the Institutional Animal Care and Use Committee at the Sahmyook University (Approval ID: SYUIACUC2013-010), and experiments were conducted according to institutional guideline.

### Materials

We purchased 1,2-dimyristoly-*sn*-glycero-3-phosphocholine (DMPC), 1,2-dipalmitoyl-*sn*-glycero-3-phosphocholine (DPPC), 1,2-distearoyl-*sn*-glycero-3-phosphocholine (DSPC), 1,2-distearoyl-*sn*-glycero-3-phosphoethanolamine-*N*-[methoxy(polyethylene glycol)-2000] (DSPE-PEG), and cholesterol from Avanti Polar Lipid, Inc. (Alabaster, AL). Chemically-synthesized ELP, [VPGVG]_n_, and ELP-lipid conjugates were provided by Peptron, Inc. (Daejeon, Korea). Doxorubicin (DOX) and calcein were purchased from Sigma-Aldrich (USA).

### Phase transition temperature of ELP-lipid conjugates

The transition temperatures of ELP-lipid conjugates were identified by measuring transmittance at 280 nm in the temperature range of 20–55°C, using a scan rate of 1°C per minute, in triplicate. The ELP-lipid conjugates were expressed as SA-Vn. The lipid moiety, stearyl group, was introduced to N-terminus of ELP peptide by amide bond and abbreviated to SA. The ELP (VPGVG) unit and repeating number were considered as Vn. The C-terminus of ELP was amidated to control the physical property of ELP- lipid conjugates. The details of ELP-lipid conjugate were summarized in Table 1. The transmittance profiles were checked for different concentrations of ELP-lipid conjugates from 0.1mM to 1 mM in phosphate-buffered saline (PBS) buffer. The data were collected on a Cary 300 Bio UV-visible spectrophotometer equipped with a multi-cell thermoelectric temperature controller from Varian (Palo Alto, CA). The transition temperature (Tt) was considered to be the temperature corresponding to the maximum of the first derivative of transmittance versus temperature.

**Table 1:** List of ELP-lipid conjugates.

ELP-lipid conjugate	Sequence
SA-V2	Stearoyl-(VPGVG)_2_-NH_2_
SA-V3	Stearoyl-(VPGVG)_3_-NH_2_
SA-V4	Stearoyl-(VPGVG)_4_-NH_2_
SA-V5	Stearoyl-(VPGVG)_5_-NH_2_
SA-V6	Stearoyl-(VPGVG)_6_-NH_2_

### Preparation of DOX-loaded liposome

Liposomes were prepared using a previously reported method with minor modifications [19]. The general formulation of the liposome was phosphocholine lipid:DSPE-PEG:cholesterol:ELP-lipid conjugates = 55∶2:X:Y (molar ratio). We encapsulated small molecules, calcein or doxorubicin (DOX), according to the experiment. In summary, lipids and modified ELPs were dissolved in chloroform, and the solvent was removed under reduced pressure. The thin film of lipids/ELP-lipid conjugates was dispersed in the calcein solution by vortexing and sonication. The liposome suspension was extruded through a polycarbonate membrane with 100-nm pores to facilitate formation of homogeneous liposomes and was followed by removal of uncapsulated calcein with PBS by size-exclusion chromatography (SEC) using a Sepadex (G-50) column. Calcein was encapsulated into the liposome during the hydration step, after lipid film formation. In the case of DOX encapsulation, DOX was loaded into liposomes using the ammonium sulfate (250 mM) gradient method. After film formation, 250 mM ammonium sulfate solution was added and the solution was vortexed and sonicated, followed by extrusion through polycarbonate membrane with 100 nm pores. Next, the exterior buffer of the liposome suspension was exchanged with 25 mM Tris⋅HCl (pH 9.0) by size exclusion chromatography (SEC) using a Sepadex (G-50) column. DOX was added to the liposome suspension at 1∶0.2 (w/w, phospholipid:DOX), and the mixture of liposome suspension and DOX was incubated at 37°C for 1 hr. Finally, unloaded DOX was removed by SEC, using PBS, pH 7.4 as eluent. The encapsulation efficiency of DOX by liposome was estimated from the absorbance of the liposome dissolved in dimethyl sulfoxide (DMSO) at 490 nm (UV-VIS Spectrometer), before and after purification with the Sephadex (G-50) column. The loading efficiency (LE) was empirically found to be variable depending on the amount of drugs contained in the liposome solution. In case that 1 mL of liposome suspension contains more than 500 µg of DOX, 50∼60% of LE was shown; whereas, 95% of LE was obtained when 300 µg of DOX was mixed with the same volume of liposome suspension.

### Drug release from liposomes

Thermosensitive drug release was measured using the fluorescent quenching property of calcein or DOX. Each aliquot of liposome suspension was incubated in a preheated chamber for 5 min, followed by ice bath quenching. The fluorescent intensity was monitored at 520 nm (for calcein) or 615 nm (for DOX), with excitation at 490 nm, by a fluorescence spectrometer (PerkinElmer, Envision 2104-multilabel reader). The drug release percentage was calculated according to the following equation: (*F_t_ -F_i_*)/(*F_f_ -F_i_*) ×100%, where *F_t_* and *F_i_* denote the fluorescent intensities of the heated and initial (at room temperature) liposome suspension, respectively. Here, *F_f_* is the fluorescent intensity of the liposome solution after addition of 1% TritonX-100 containing ethanol to disrupt the liposome completely.

### Size and morphology of liposomes

The size of liposome was measured by dynamic light scattering (DLS) at 25°C using a Zetasizer Nano ZS (Malvern instrument, UK) with a He-Ne laser at a wavelength of 633 nm and a detection angle of 90 degrees.

Liposome morphology was observed using a cryo-transmission electron microscope (cryo-TEM). Samples for cryo-TEM were prepared on carbon film-supported grids with holes. Thin aqueous film, blotted with filter paper, was fabricated by Vitrobot (FEI) and immediately plunged into liquid ethane. The resulting grids were stored in liquid nitrogen and transferred to a cryotransfer holder (Gatan). Images were obtained using a CCD camera (2 k, Gatan) with a Tecnai F20 field emission gun electron microscope operated at 200 kV (FEI) in low dose mode.

### Molecular dynamics simulations of lipid bilayers

All coarse-grained (CG) molecular dynamics (MD) simulations and analyses were performed using the GROMACS4.5.5 simulation package [20]–[22] with the “MARTINI” CG force field (FF) [23]–[25], which lumps 3 or 4 heavy atoms into each CG bead. Models for DPPC, DSPE-PEG, cholesterol, and ELP were taken directly from the MARTINI FF. All simulated systems are listed in Table 2. For the system with ELP molecules, ELPs of three different lengths ([VPGVG]_n_, where n = 1, 2, and 3) were simulated.

**Table 2:** List of all simulations.

No. of molecules				Molar ratio
DPPC	DSPE-PEG	Cholesterol	ELP	
1296	48	360	0	55∶2∶15∶0
1296	48	360	6	55∶2∶15∶0.25
1296	48	0	10	55∶2∶0∶0.41
1296	48	240	10	55∶2∶10∶0.41
1296	48	360	10	55∶2∶15∶0.41

The lipid bilayer, which consists of DPPC, DSPE-PEG, cholesterol, and ELP molecules at different molar ratios, was solvated with ∼35,000 CG water molecules (representing ∼140,000 real water molecules) in a periodic box of size 20×20×16 nm^3^. Since a single DSPE-PEG has a net charge of −1, 48 counterions (Na^+^) were added to neutralize the bilayer system. A cutoff of 12 Å was used for Lennard-Jones (LJ) and electrostatic interactions. The LJ and Coulomb potentials were shifted to 0 between 9 and 12 Å, and between 0 and 12 Å, respectively. Since the transition temperature for MARTINI DPPC is 295 K [26], which is ∼20 K lower than the experiment value, a temperature of 295 K and a pressure of 1 bar were maintained by applying the Berendsen thermostat and barostat in the NP_xy_P_z_T ensemble [27]. Simulations were performed for 2 µs with a time step of 8 fs, which is lower than the typical time step of 20–40 fs because of the inclusion of PEG dihedral potentials. The last 500 ns were used for analyses.

### Animals and tumor implantation

Six-week-old male BALB/c nude mice were used in this study. To produce tumors, the murine mammary tumor cell line, EMT-6, was cultured in RPMI media (Sigma-Aldrich Co. St Louis, MO) supplemented with 10% fetal bovine serum and 1% antibiotics. For *in vivo* injections, EMT-6 cells were trypsinized and centrifuged at 1,200 rpm for 3 min at 4°C, washed twice with PBS, and reconstituted in Dulbecco’s phosphate-buffered saline (D-PBS; Gibco, Carlsbad, CA). EMT-6 cells (1.0×10^6^ cells/50 µL of D-PBS) were injected subcutaneously into both thigh areas.

### Drug accumulation and antitumor efficacy study

Tumor-bearing mice (n = 4) were anesthetized by ventilation with isoflurane (Forane; Baxter, Deerfield, IL) and fixed on a custom-made holder. The temperature in the water bath was maintained at 42°C by a thermostat (NTT-2200, Eyela, Tokyo, Japan).

These experiments were carried out when the average tumor volume was approximately 500 mm^3^ (300–350 mg in mass, ±10%). The tumor tissues of mice were initially preheated for 30 min using a water bath. After removal from the water bath, the drugs (5 mg/kg of free DOX or its equivalent) were administered through the tail vein of mice in the DOX and DOX-encapsulated liposome groups. The same volume of PBS was injected intravenously to mice in the control group. At different time points (1, 6, and 12 hr) after drug administration, mice were anesthetized and sacrificed for tumor tissue collection. Tumor tissues isolated from mice were dissociated in nuclear lysis buffer composed of 0.25 mol/L sucrose, 5 mmol/L Tris-HCl, 1 mmol/L MgSO_4_, 1 mmol/L CaCl_2_ (pH 7.6) by a homogenizer. After the addition of 10% (v/v) Triton X-100, the sample was then centrifuged for 10 min at 12,000×g and the supernatant was loaded onto a 96-well plate for determination of fluorescence at Ex 490 nm/Em 615 nm by a fluorescence spectrometer (PerkinElmer, Envision 2104-multilabel reader). DOX concentration was obtained by comparing fluorescence with a calibration curve generated from known amounts of DOX in tumor tissue homogenates, and the amount of DOX accumulated from free DOX and the liposome group was determined as µg doxorubicin/g tissue.

For detecting intratumoral distribution of DOX, 8 mg/kg of the drug was introduced to reinforce the visibility of DOX accumulated in tumor tissues. Tumor tissues were collected at 6 hr-post injection of drugs, embedded immediately in OCT compound, and frozen in liquid nitrogen. Upon the histological analysis, samples were cryosectioned as 10 µm tissue sections and imaged by a confocal laser scanning microscope (LSM710, Carl Zeiss). Subsequent to imaging of DOX, tumor tissue sections were fixed in acetone for 10 minutes, stained with anti-CD31 (MAB1398Z, Millipore) and Hoechst (Sigma-Aldrich), and mounted in Vectashield mounting medium (Vector Laboratories).

The maximized drug accumulation was shown in 6 hrs after drug administration among the different time points (1, 6, and 12 hrs). We chose this time course for the anti-tumor efficacy study, and compared with free DOX and control groups in the presence/absence of mild hyperthermia. Briefly, the tumor tissues of mice were initially preheated for 30 min as described above and the DOX-loaded liposome or free DOX (5 mg of DOX/kg of mouse) was administered through the tail vein of mice. 6 hrs of lag-time was given to obtain the maximized accumulation of drug-encapsulated liposomes in the tumor tissues. Subsequently, the tumor tissues of mice were stimulated with another heating treatment for 30 min using a water bath in order for bursting-out of drugs from the thermosensitive liposomes accumulated in the tumor tissues. The tumor size was measured on day 0, 1, 2, 3, 5, 7, 9, 11, 13, and 15 by vernire calipers, and the volume was calculated by the following formula: Volume  =  (width of tumor)^2^× (length of tumor) × π/6.

### Statistical analysis

The data obtained in our anticancer efficacy study was analyzed statistically with two-tailed unpaired *t*-test using a one-way analysis of variance (ANOVA) test implemented by SigmaPlot 11 software.

## Results

### Concept design of ELP-incorporated thermosensitive liposome (e-TSL)

**Figure 1:**
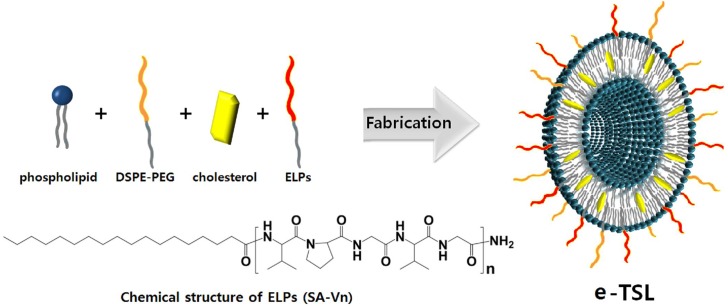
Schematic diagram of e-TSL and the chemical structure of ELP-lipid conjugates.

To develop the TSL under mild hyperthermia (39–42°C), we introduced a short chain elastin-like polypeptide modified with lipid moieties (ELP-lipid conjugate). An elastin-like polypeptide (ELP) consisting of a [VPGXG]n pentapeptide repeat, where X can be any amino acid except proline, possesses thermosensitivity and a transition temperature that is adjustable according to length, sequence, concentration, pH, and ionic strength [15]–[18]. The mechanism of this phenomenon is known: a hydrogen bonding interaction between ELP and a water molecule dominates in aqueous solution below the transition temperature (Tt), while an intramolecular ELP hydrophobic interaction is predominant in aqueous solution above the Tt, resulting in a conformational change from a random coil to a β-turn. In the present study, we utilized ELP as a heat-triggered moiety, and a single hydrocarbon tail (stearyl group) was conjugated at the N-terminus of ELP for incorporation into the bilayer of the liposome. The expected ELP-liposome hybrid vesicle is depicted in Figure 1.

### ELP-lipid conjugates screening

**Figure 2:**
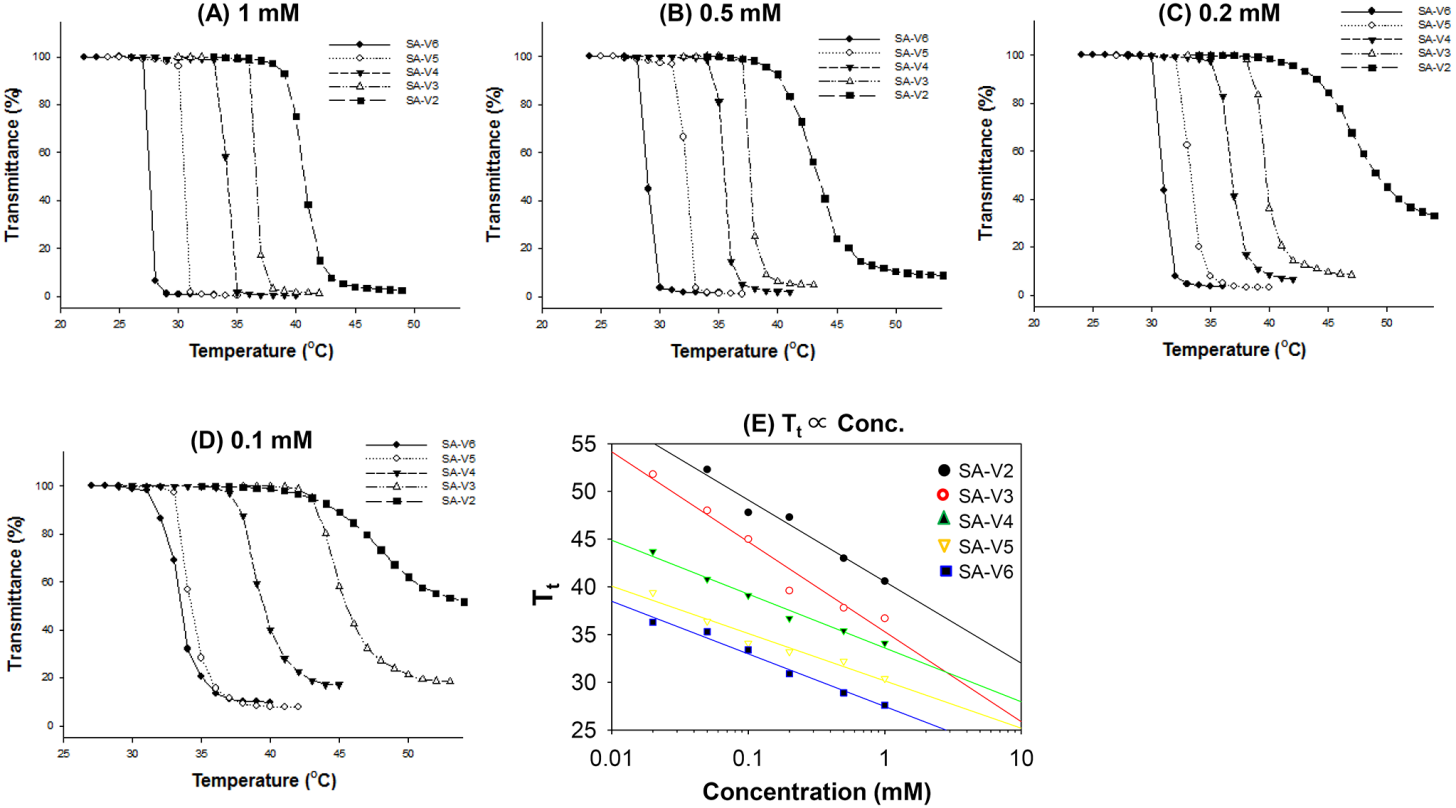
Transmittance profile of ELP-lipid conjugates of (A) 1 mM, (B) 0.5 mM, (C) 0.2 mM, and (D) 0.1 mM in PBS at pH 7.4., and transition temperature as function of the concentration (mM) of various ELP-lipid conjugates (E). The turbidity of ELP-lipid conjugates were characterized by monitoring transmittance at 280 nm as a function of temperature. Solution of ELP-lipids conjugates were heated at a constant rate of 1°C/min. The transition temperature (Tt) was defined as the temperature at which the solution of ELP-lipid conjugate reached 50% of transmittance. Data is shown as mean ± S.D. (n = 3).

ELPs are smart biopolymers, which exhibit reversible precipitation above a certain temperature, called inverse phase T_t_. We analyzed the T_t_ of modified ELPs using UV spectrometry. ELPs, thermally sensitive polypeptides with a repeating unit (VPGVG), undergo an inverse temperature phase transition above a certain Tt. Depending on the concentration and the length of the repeating unit (length of peptide), the ELP shows unique features of intermolecular interaction. We prepared five ELP-lipid conjugates with different ELP lengths and identified their T_t_, according to the concentration and the number of repeating units. Figure 2 shows the transmittance of ELP-lipid conjugates as a function of temperature and concentration. The phase transition of the ELP-lipid conjugate was observed in all groups undergoing a change of turbidity. Depending on the number of repeating units (the length of ELP) and the concentration, we observed different phase transition behaviors. In the case of a longer ELP-conjugated lipid, a sharp phase transition was observed, whereas a relatively short ELP-conjugated lipid showed a somewhat smooth phase transition. Tt decreased as concentration increased and the sequence became longer, as expected. This is most likely because the intermolecular hydrophobic interaction of ELPs is readily occurred even at the lower temperature. We also confirmed that the temperature-responsive behavior of ELP was maintained, even after modification of the N-terminal peptide.

### Drug release study dependent on liposome formulation

We designed the e-TSL composed of four different components, phospholipid, cholesterol, DSPE-PEG, and ELP-lipid conjugates. The temperature-dependent release profile of liposome was investigated using calcein and DOX, which is fluorescent and easy to detect upon release from the liposome.

#### The effect of main lipid

**Figure 3:**
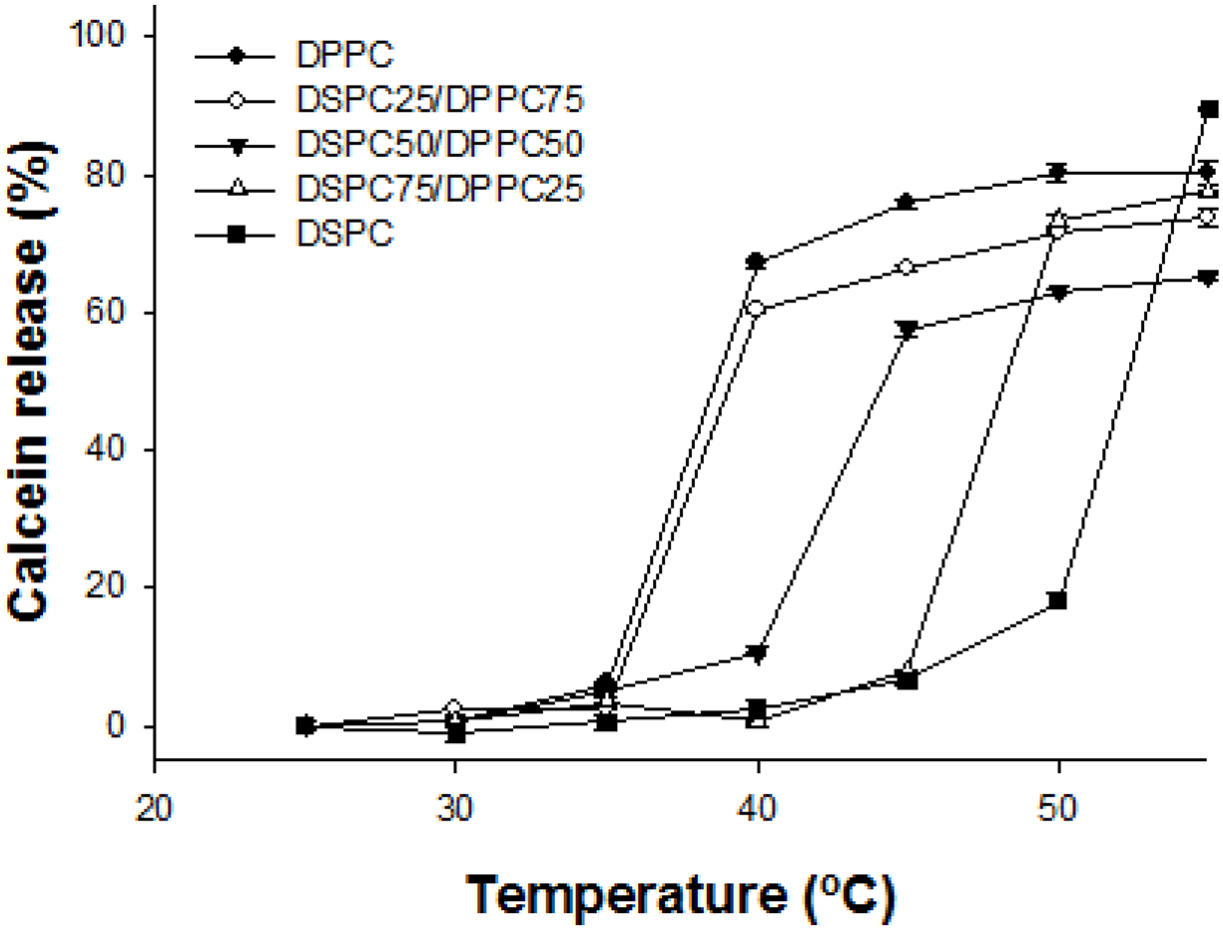
Calcein release profile of the liposome with the formulations of varied phospholipid compositions. Phospholipid (DPPC/DSPC = 100-X/X):DSPE-PEG:cholesterol:SA-V6 = 55∶2∶10∶0.55 (X = 0, 25, 50, 75, and 100). Samples were measured after 5 min incubation at desired temperatures from 25 to 55°C by fluorometry at Ex. 493 nm/Em. 513 nm. Data is shown as mean ± S.D. (n = 3).

We prepared five liposomes with different ratios of DPPC and DSPC, in order to investigate the effect of the main lipid on temperature-responsive release of liposomes. The liposomes were formulated with phospholipid (DPPC, DSPC, and DPPC/DSPC), DSPE-PEG, cholesterol, and SA-V6 (phospholipid:DSPE-PEG:cholesterol:SA-V6 = 55∶2∶10∶0.55). Figure 3 shows that while T_t_ increased as the ratio of DPPC decreased, it remained lower than the original melting temperature of DSPC because of the other elements (DSPE-PEG, ELP, and cholesterol). Compared to other liposomes with different formulations, liposomes composed of DPPC and DPPC with 25% DSPC showed similar calcein leakage profiles.

#### Cholesterol effect

**Figure 4:**
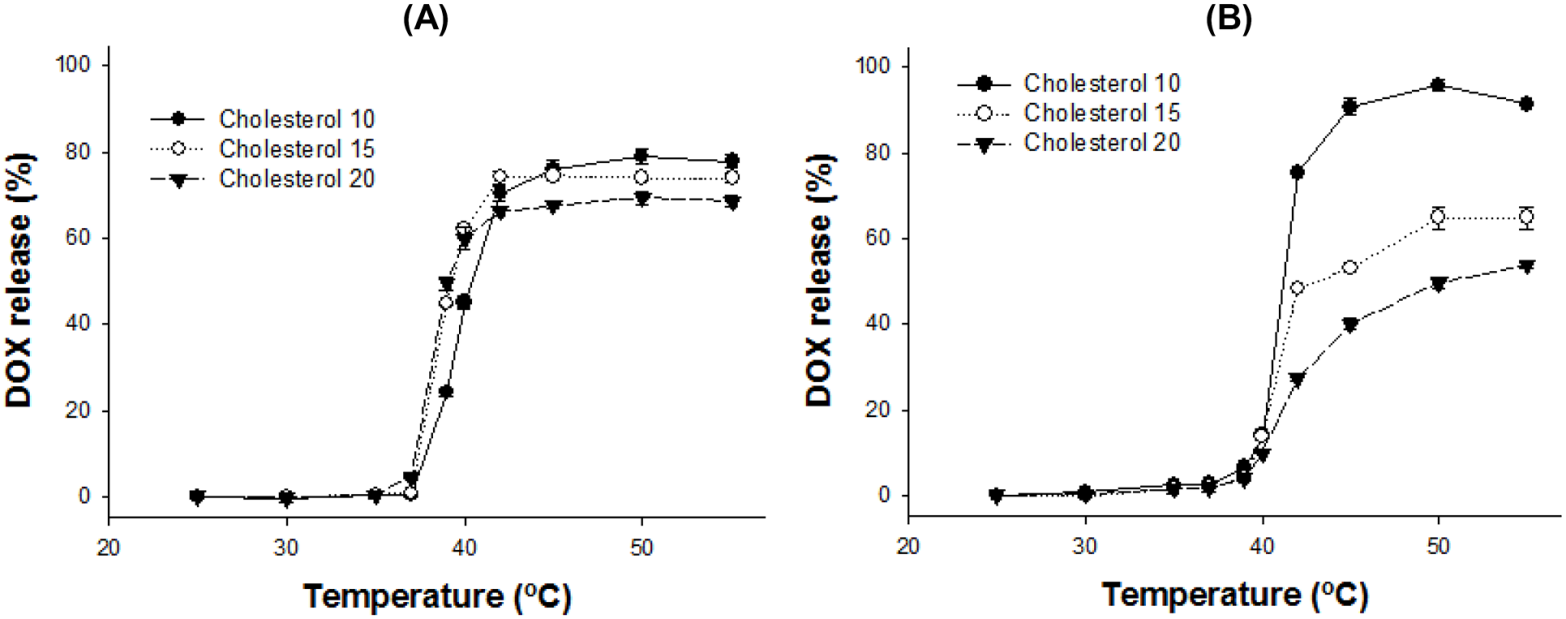
DOX release profile of the liposome with the formulations. (A) DPPC:DSPE-PEG:cholesterol:SA-V3 = 55∶2:X:55 and (B) DPPC/DSPC (75/25):DSPE-PEG:cholesterol:SA-V3 = 55∶2:X:55 (X = 10, 15, or 20). The amounts of DOX release were measured after 5 min incubation at desired temperature from 25 to 55°C by fluorometry at Ex. 490 nm/Em. 615 nm. Data is shown as mean ± S.D. (n = 3).

Next, we verified the effect of cholesterol on the release profile. It is well known that cholesterol is a major factor of membrane stability and fluidity. The release profile of 6 different liposomes with different ratios of cholesterol was identified. As expected, the release of encapsulated molecules was delayed by increasing the ratio of cholesterol. Additionally, the maximum released amount was lower in accordance with higher cholesterol content (Figure 4).

#### ELP effect

The length of ELP, based on the repeating unit (VPGVG), caused variations in the release profile. With a shorter ELP-lipid incorporated liposome, the T_t_ of release from the liposome was increased. It correlated well with the trends of reverse T_t_ of ELP, depending on the length of peptide and temperature. Presumably, because of the destabilization of the liposome membrane induced by the conformational change of ELP from random coil to β-turn, the encapsulated molecules are released from the liposome. With the longer ELP, the T_t_ of the liposome is lower than that of the liposome with the shorter ELP (data not shown).

**Figure 5:**
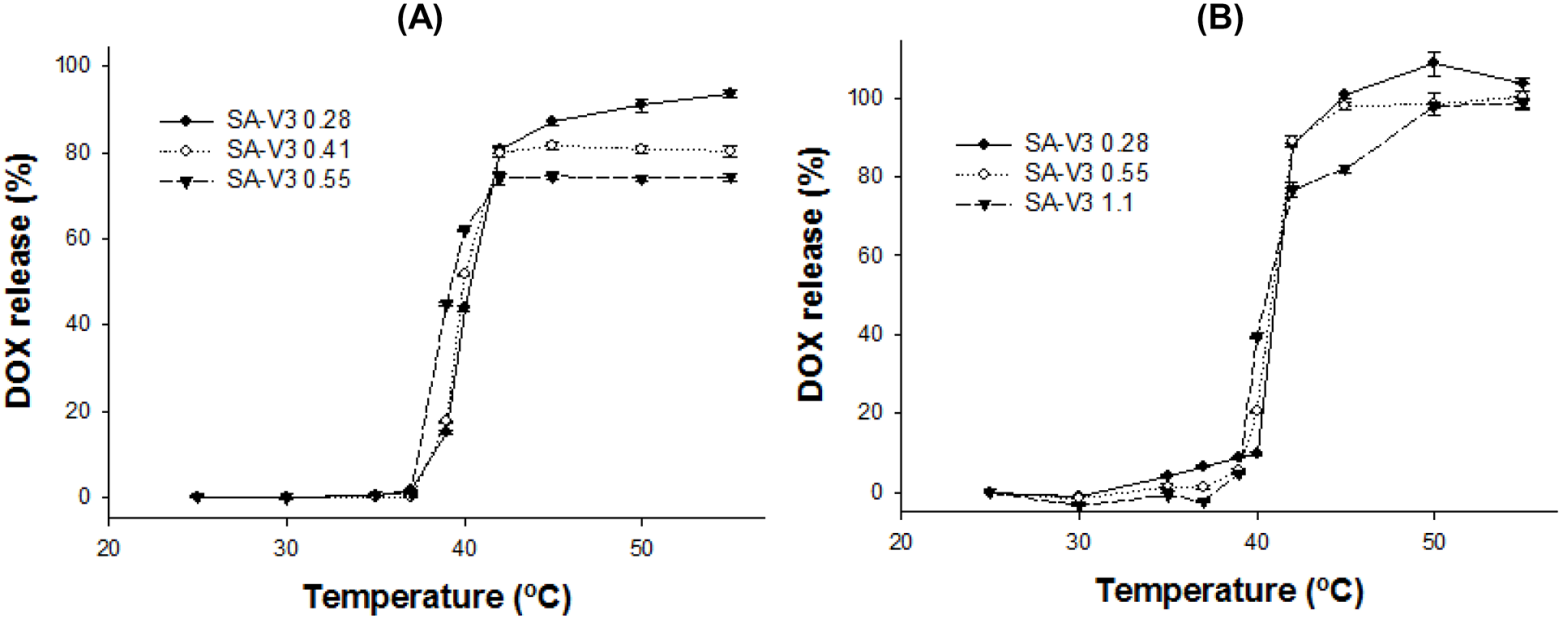
DOX release profile of the liposome with the formulations. (A) DPPC:DSPE-PEG:cholesterol:SA-V3 = 55∶2∶15:Y_1_ (Y_1_ = 0.28, 0.41, or 0.55) and (B) DPPC/DSPC (75/25):DSPE-PEG:cholesterol:SA-V3 = 55∶2∶10:Y_2_ (Y_2_ = 0.28, 0.55, or 1.1). The amounts of DOX release were measured after 5 min incubation at desired temperature from 25 to 55°C by fluorometry at Ex. 490 nm/Em. 615 nm. Data is shown as mean ± S.D. (n = 3).

The role of ELP was investigated as a heat trigger in this liposome system. The amount of DOX release from the liposome was determined by ELP above 37°C. According to the formulation, the DOX release was different from about 70 to 100% over mild hyperthermic temperature (Figure 5). There was an inverse relationship between T_t_ and the amount of ELP for drug release: the higher the amount of ELP, the lower the T_t_. At the same time, the liposomes with higher percentage of ELP showed ∼40% of drug leakage below 40°C (Figure 5).

### Optimized liposome formulation for mild hyperthermia

**Figure 6:**
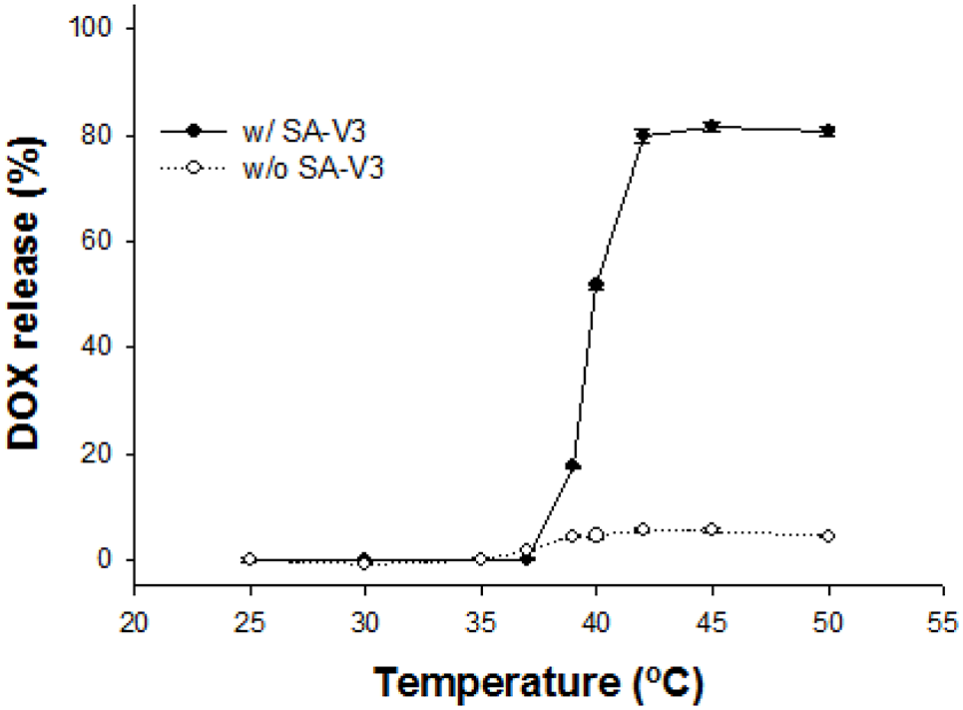
DOX release profile of the liposome with the formulations. DPPC:DSPE-PEG:cholesterol = 55∶2∶15 in the presence/absence of SA-V3 (molar ratio∶0.41). The amounts of DOX release were measured after 5 min incubation at desired temperature from 25 to 55°C by fluorometry at Ex. 490 nm/Em. 615 nm. Data is shown as mean ± S.D. (n = 3).

Generally, the temperature range of clinically relevant mild hyperthermia is from 39–42°C. The first requisite of TSL for mild hyperthermia is a very sharp drug release within 3–4°C. Formulation screening for e-TSL identified the DPPC only or DPPC/DSPC (75/25) as a main lipid, cholesterol as a stabilizer, and SA-V3 as a trigger. With the 3 factors for thermosensitive peptide-incorporated liposome, further optimization studies were carried out to refine and improve the performance of liposome. As we already identified, the observed maximum drug release percentage at 42°C decreased by increasing the mol% content of cholesterol. In most of the tested liposome formulations, the influence of SA-V3 on temperature dependent drug release at 42°C was observed and it was more than 80% within 5 minutes. With higher amounts of SA-V3, the temperature sensitivity for drug release increased. However, drug leakage in the temperature range of 37–39°C occurred as the mol% contents of SA-V3 increased, especially for the DPPC liposome. By considering the effect of cholesterol and SA-V3, the optimal formulation was finalized as the liposome composed of DPPC/DSPE-PEG/cholesterol/SA-V3 ( = 55/2/15/0.41) for stable blood circulation and effective drug release under mild hyperthermia. Actually, the liposome composed of DPPC showed phase transition under hyperthermia, even without ELP. To confirm the effect of ELP as a trigger on a screened formulation, liposomes were prepared with or without SA-V3, and their drug release profiles were checked (Figure 6). As shown in Figure 6, the liposome in the absence of SA-V3 showed a drug release of less than 10% of encapsulated DOX under mild hyperthermia. However, the maximum amount of drug release and the temperature sensitivity of the ELP-free liposome were much lower than that of the ELP-incorporated liposome.

### Size and morphology of e-TSL

**Figure 7:**
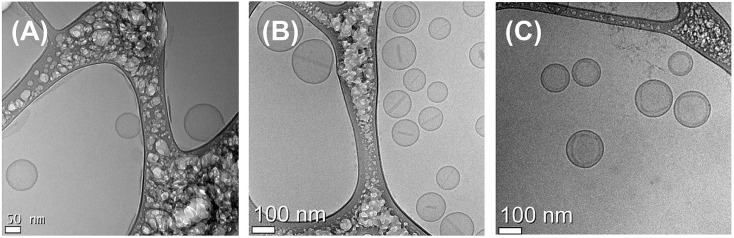
Cryo-TEM images with varying amounts of DOX in the liposome. DPPC:DSPE-PEG:cholesterol:SA-V3 = 55∶2∶15∶0.41, with (A) liposome only, (B) 300 µg DOX/mL and (C) 500 µg DOX/mL.

**Figure 8:**
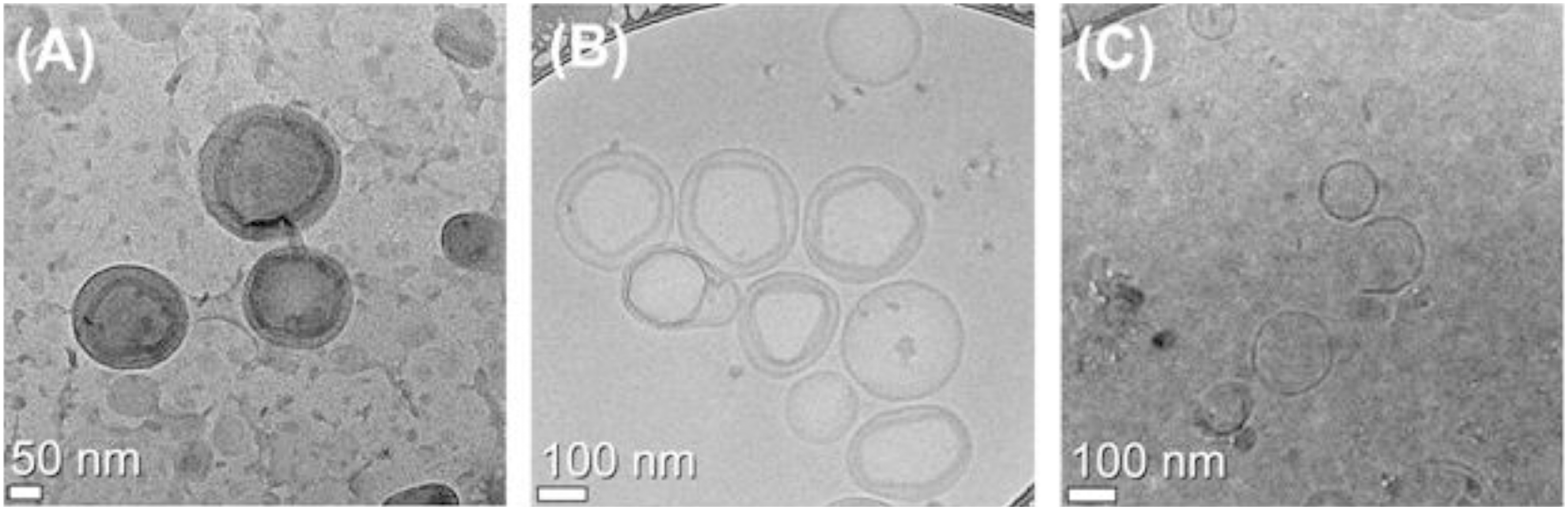
Cryo-TEM images of the liposome at (A) 25°C, (B) 37°C, and (C) 42°C. Liposomes were composed of the formulation DPPC:DSPE-PEG:cholesterol:SA-V3 = 55∶2∶15∶0.41 and heat-treated for 5 min at indicated temperature in advance and followed by general cryo-TEM measurement.

With the optimized ELP-incorporated liposome, the size and morphology of the liposomes were identified. The diameter of the liposome was 161±4 nm and polydispersity index (PdI) value was relatively good (less than 0.05). To visualize the DOX-encapsulated liposome, transmission electron microscopy (TEM) was utilized. TEM is a powerful tool for the structural analysis of nano-sized materials. It clearly showed round-shaped liposomes, and visualized the presence of DOX as an electron-opaque band, which was loaded into the liposome by ammonium sulfate gradient procedures. The size of liposomes visualized by TEM corresponded to the DLS results. Liposomes with varying contents of DOX had different distributions in the TEM images (Figure 7). Encapsulated DOX was bundled with relatively low loading amounts of DOX. However, encapsulated DOX became looped in liposomes with a high content of DOX. The mean size of each liposome determined by DLS was 171±3 nm (bundle type type) and 177±2 nm (loop type). We also observed structural changes of DOX-encapsulated liposomes at 25, 37, and 42°C. The TEM images indicated drug encapsulation and release from the liposome. As we had already confirmed, over 80% of the encapsulated amount of DOX in the liposome was released at 42°C. The looped form of DOX disappeared in the image obtained for the liposome group incubated at 42°C (Figure 8).

### Molecular dynamics simulations of lipid bilayers

**Figure 9:**
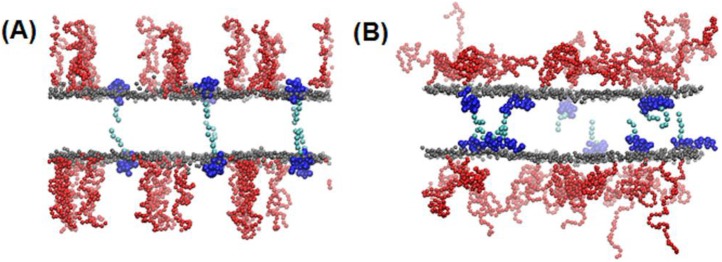
Snapshots at the (A) beginning (0 ns) and (B) end (2 µs) of the simulation of the bilayer system. The molar ratio of liposome formulation is DPPC:DSPE-PEG:cholesterol:SA-Vn 55∶2∶15∶0.41. Gray, red, dark-, and light-blue colors respectively represent the lipid-head phosphate, PEG, ELP head (peptide), and tail (carbon chain) groups. For clarity, lipid tail, water, and ion beads are omitted. The images were created with Visual Molecular Dynamics [29].

Lipid bilayers, which consist of DPPC, DSPE-PEG, cholesterol, and ELP-lipid conjugates (SA-Vn, n = 1, 2, and 3), were simulated at 5 different molar concentrations of cholesterol and ELP for 2 µs. Figure 9 shows snapshots from the beginning and end of the simulation (at the molar ratio of liposome formulation, DPPC:DSPE-PEG:cholesterol:SA-Vn = 55∶2∶15∶0.41). The peptide chains of ELP, which were initially positioned on the bilayer surface, insert into the bilayer because of the interaction between hydrophobic amino acids and lipid tails, as observed in our previous all-atom simulations [28]. The xy-plane areas (the bilayer surface) and energies for each system reach steady-state values within 1.5 µs, indicating that the simulations are equilibrated within the simulated time scales (Supplementary data, Figure S1). Note that our previous all-atom simulations showed that ELPs in water become more collapsed and folded as temperature increases, while those in lipid bilayers become linearly swollen with mostly random conformation at increased temperature, indicating the structural difference of ELPs in aqueous and membrane environments. This implies that the bilayer may be disrupted more effectively by extended random coils of ELPs than by the compact ELPs.

**Figure 10:**
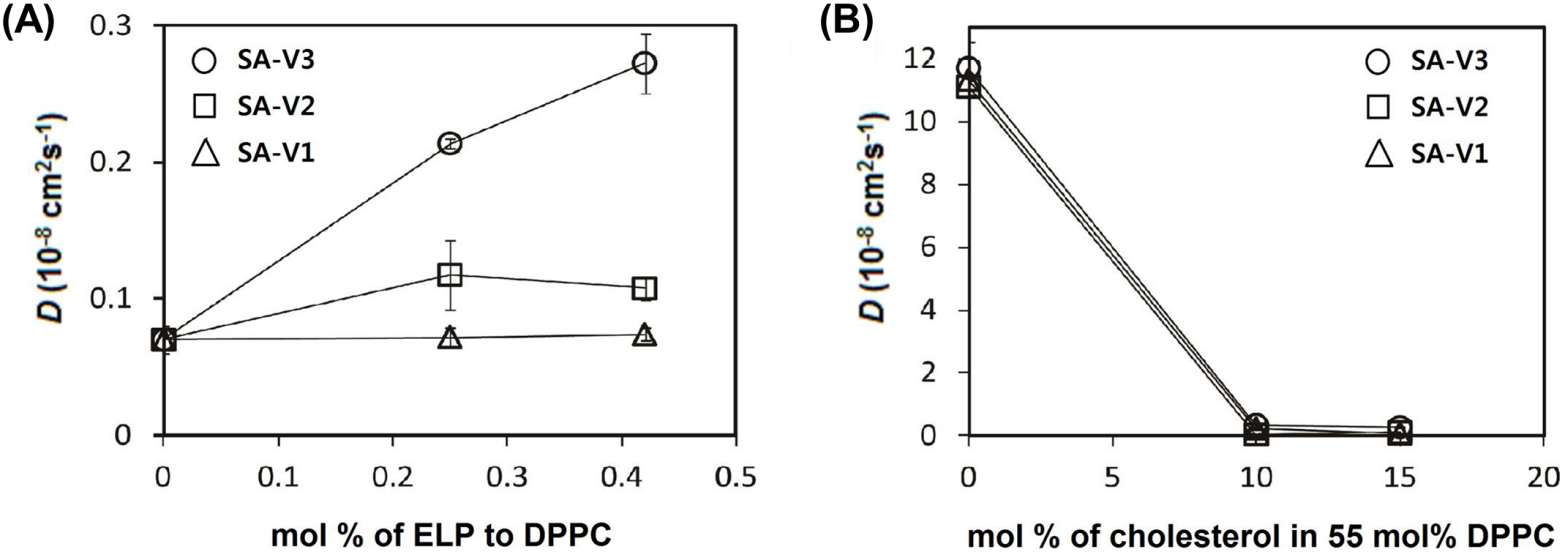
Average lateral diffusion coefficients (*D*) of DPPC lipids. *D* was calculated from the slopes of the mean-square displacements in the xy-plane (the direction perpendicular to the bilayer normal), for the simulation systems with different ELP lengths (SA-V1, -V2, and V3) as functions of the molar ratio of (A) ELP and (B) cholesterol.

Experiments have shown that the lateral mobility of lipids significantly depends on the bilayer phase, indicating that the phase of lipid bilayers can be characterized by calculating lateral diffusion coefficients of lipids [30], [31]. Since the MARTINI FF has successfully predicted the experimentally observed lateral diffusivities of DPPC lipids at different phases [26], we calculated lateral diffusion coefficients (*D*) of lipids from the slopes of the mean-square displacements (MSD) of lipids as a function of time. Figure 10 shows that *D* drastically increase at higher concentrations of SA-V3, indicating an effect of ELP on the lipid mobility, presumably because the inserted ELP disorders the bilayer, as observed in our previous all-atom simulation. SA-V1 and SA-V2, which consist of shorter ELPs, show that lateral diffusivities do not change much at different ELP concentrations, indicating that shorter ELPs do not significantly increase the lipid dynamics. This implies that lipids are more disordered by longer ELPs than by shorter ones, and thus the lipid bilayer with shorter ELPs should have the higher Tt, consistent with our experimental observation of higher Tt of the liposome with shorter ELP chains. For the effect of cholesterols, lateral diffusion coefficients were significantly reduced in the presence of cholesterol, indicating that cholesterol inhibits the dynamic motion and permeation of the bilayer. This effect of cholesterol on lipid dynamics is not influenced by the ELP length.

### *In vivo* drug accumulation and antitumor efficacy study

**Figure 11:**
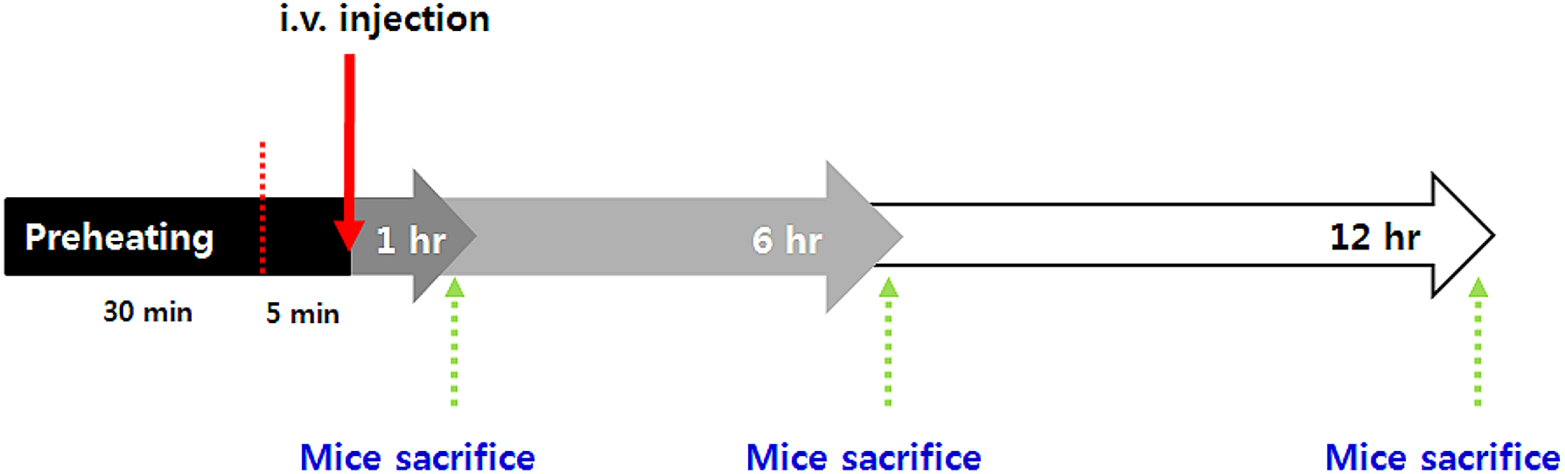
The protocol of the drug accumulation study with e-TSL-DOX and free DOX, administered under mild hyperthermia.

**Figure 12:**
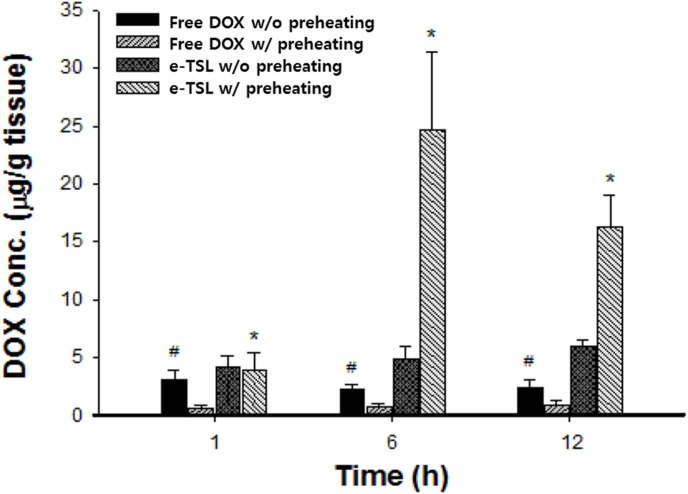
DOX accumulation in the tumor at 1, 6, and 12 hr after i.v. injection combined with preheating. *, *p*<0.005, #, *p*<0.001, significant difference compared to e-TSL without preheating and free DOX with preheating.

**Figure 13:**
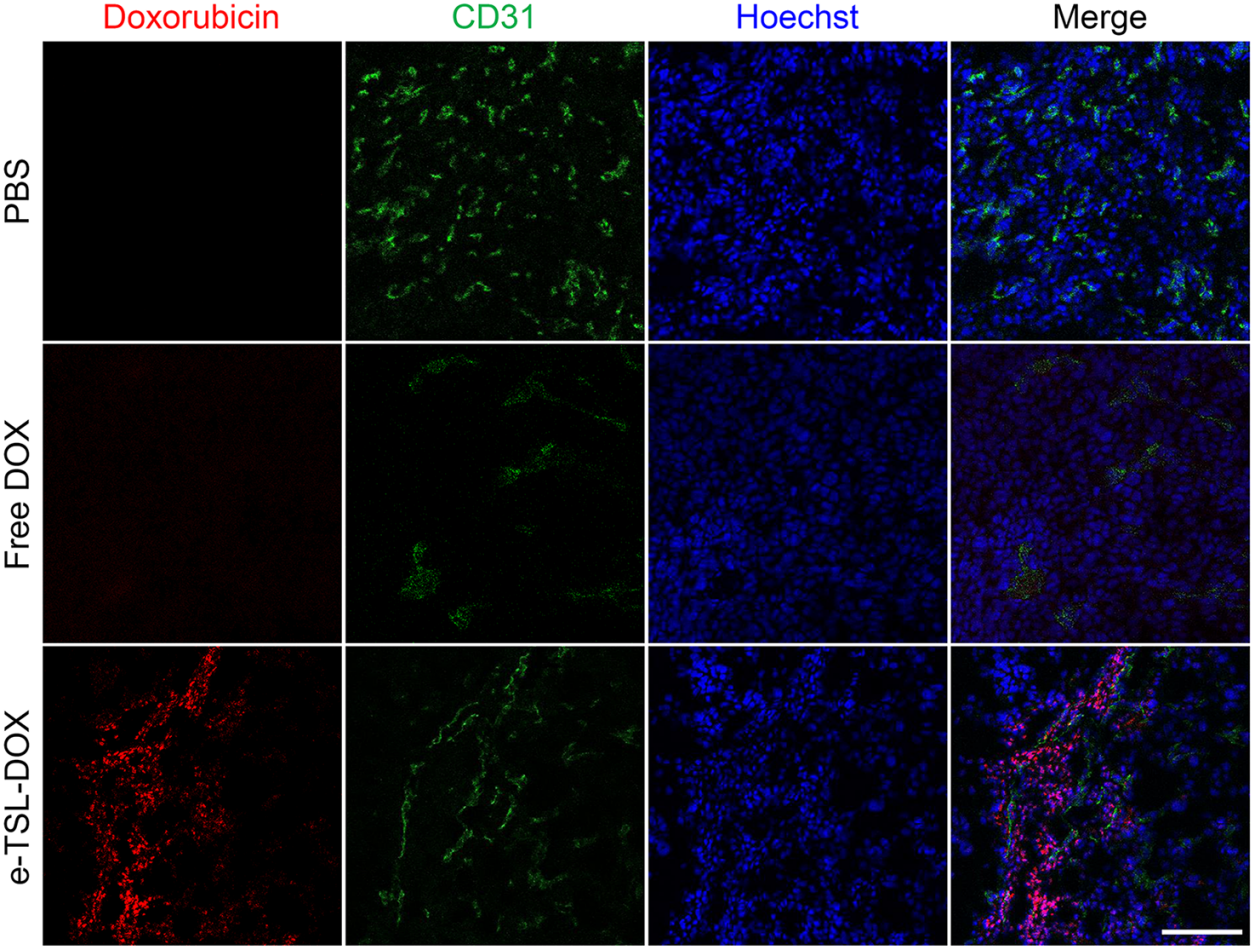
Intratumoral distribution of DOX. Red, DOX; green, CD31 (endothelial marker); blue, Hoechst (nuclear marker). Scale bar, 100 µm.

To investigate DOX delivery *in vivo*, we carried out a drug accumulation study in the tumor. The available drug amount in a tumor site is closely related with antitumor efficacy. We confirmed that the blood circulation of the DPPC-based liposome decreased gradually with time, and that the half-life of the DOX-encapsulated liposome *in vivo* was about 2.5 hr, as shown in a previous study [19]. Because tumors have inherently leaky vessels, nano-sized particles are easy to accumulate by EPR. In this study, we induced enhanced EPR by locally heating (42°C) the tumor site for 30 min before i.v. injection; the detailed procedures are described in Figure 11. As shown in Figure 11, we identified drug accumulation in the tumor at 1, 6, and 12 hr time points after injection (Figure 12). Compared to the free DOX-treated group, liposomal DOX (e-TSL-DOX) showed better accumulation in tumors at every time point with or without preheating. In particular, the group of e-TSL-DOX with preheating showed significant drug accumulation at 6 hr after i.v. injection; the difference was 5 fold with e-TSL without preheating, 11 fold with free DOX without preheating, and 31 fold with free DOX with preheating. In case of free DOX, the group without preheating resulted in more drug accumulation than its preheated group contrary to e-TSL group. To support the drug accumulation data, the histological observation of intratumoral distribution of doxorubicin was carried out. The significant fluorescence of DOX was detected only in the group of e-TSL-DOX with preheating (6 hr lag time), which was in alignment with aforementioned drug accumulation results (Figure 13).

**Figure 14:**
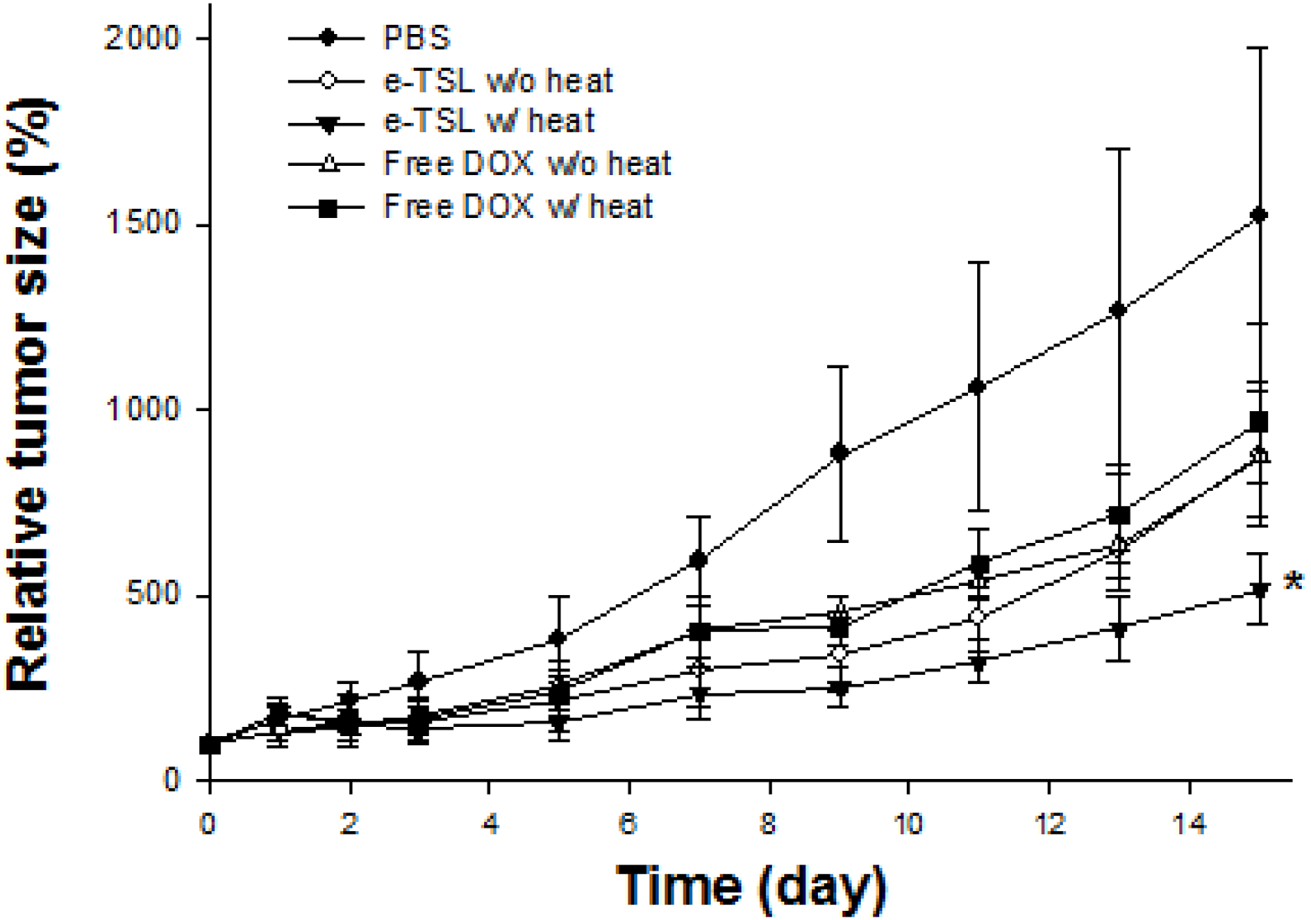
Antitumor efficacy of e-TSL and free DOX in the presence/absence of mild hyperthermia. e-TSL and free DOX were administered (5 mg DOX/kg) into tumor bearing BALB/c mice 5 min after preheating (30 min of water bath) and followed by mild hyperthermia (42°C, water bath) after 6 hrs. *, *p*<0.05, significant difference compared to free DOX and PBS (control).

Finally, we evaluated the antitumor efficacy of e-TSL with optimized hyperthermia protocol based on the results of drug accumulation study (preheating and 6 hr lag time). In the presence/absence of the mild hyperthermia, the tumor regression effect of e-TSL is shown in Figure 14. The mice group treated with e-TSL showed better antitumor efficacy than other groups and delayed tumor growth, especially with mild hyperthermia. The trend of antitumor effect was similar with the result of drug accumulation study. The more doxorubicin was accumulated, the more effective antitumor efficacy was outcome.

## Discussion

For an ideal antitumor treatment, enhanced drug delivery at the target site with minimized toxicity at off target sites, the TSLs have to be stable in circulating blood (at 37°C) and burst-released as soon as they are exposed to mildly hyperthermic temperatures. The most clinically-advanced TSL, lysolipid-containing temperature sensitive liposome (LTSL), was developed by Needham and Dewhirst and is now under clinical trials. LTSL is formulated with lyso-phospholipid, DSPE-PEG, and DPPC. Lysolipid has only one acyl chain, and tends to form a micelle structure. Due to these characteristics, lysolipid induces stabilization of grain boundaries, which are formed between the solid and liquid domain of a liposome upon heating. Rapid drug release from the LTSL occurred through lysolipid-stabilized pores when mild hyperthermia was applied. Despite the high responsiveness to heating, LTSL was reported to have the low stability of LTSL in *in vitro* serum-containing media (37°C) and *in vivo* blood circulation [32], [33]. To realize TSL formulation that allows for both physiological stability and high responsiveness to heating, we introduced cholesterol as membrane stabilizer and ELP as heat-triggered moiety to liposome. By controlling the liposome composition of DPPC (or DSPC), cholesterol, DSPE-PEG, and ELP, the Tt and amount of drug release was tunable.

To rapidly test thermosensitivity of e-TSL, calcein fluorescent dye-loaded and various ELP-lipid conjugates incorporating e-TSLs were prepared and checked calcein release. Of these, SA-V6- loaded e-TSL exhibited the optimal release (data not shown). Even if Tt of SA-V6 itself was shown to be below 36°C (Figure 2), it was shifted above 36°C after calcein loading (Figure 3). Because calcein was added to the hydration buffer before extrusion in the liposome preparation procedure, it happened to be inserted into the lipid bilayer membrane and also encapsulated inside the inner core of liposome. The inserted calcein molecules probably interfere with the hydrophobic interaction of ELP, resulting in the shift of Tt. On the other hand, to load DOX into liposome, the remote loading method using ammonium sulfate gradient was employed, inducing the encapsulation of DOX inside the inner core as crystallized, and the encapsulated DOX did not influence on the properties of ELP conjugated lipid bilayer membrane. As shown by Figure 2E, SA-V2, SA-V3, and SA-V4 were evaluated as the relevant candidates for thermally triggered moiety under the mild hyperthermia temperature (40∼45°C). We assessed the DOX release profile of e-TSL comprising SA-V2, SA-V3, and SA-V4 (data not shown). Among these, SA-V3 was chosen for further *invitro* and *invivo* studies as highly potent profile was shown.

We found that the amount of drug release was related to cholesterol content, and the T_t_ could be finely tuned by the length/amount of ELP-lipid conjugate in the liposome. For thermosensitive liposome-based therapy under mild hyperthermia, we carefully analyzed and selected the formulation after screening. To avoid large leakages of the encapsulated drug during blood circulation, a higher concentration of cholesterol was necessary for a stable liposome. The T_t_ of drug release was increased with incorporation of a minor amount of ELP. Taken together, the balance between the two factors, cholesterol and ELP-lipid conjugate, was important for preparation of a certain-temperature sensitive liposome formulation. When the amount of cholesterol in a tested formulation was more than 10 mol%, drug release at 42°C was less than 70%. While the addition of more ELP-lipid conjugate in the formulation could compromise maximum drug release, drug leakage was observed at 37–39°C. Drug leakage is thought to be mainly dependent on the amount of cholesterol, but is partly affected by relatively a higher incorporation of ELP-lipid conjugate (>1.1 mol%). The effect of ELP-lipid conjugate on the drug release of liposomes is not only the control of T_t_ but also of drug leakage. The findings of our simulation study compared favorably with our experimental observations, which showed that the extent of DOX or calcein release increased at a higher concentration of ELP but decreased at a higher concentration of cholesterol.

The optimized liposome formulation showed somewhat long blood circulation (>2.5 hr) [15]. To maximize antitumor efficacy, with optimized formulation of e-TSL, we set up the treatment protocol combined with mild hyperthermia in mice. The advantage of tumor tissue for drug accumulation is an EPR effect, which we enhanced by heating the tumor prior to i.v. injection of the DOX-encapsulated liposome. The hyperthermia in tumors may have increased vascular permeability by widening the gaps of endothelial walls which were preserved for some time after heat treatment [34]. We utilized mild hyperthermia as the stimulus for drug release as well as a booster for the EPR effect. The optimized liposome formulation (DPPC/DSPE-PEG/Cholesterol/SA-V3 = 55/2/15/0.41) was stable enough to circulate in the blood for a relatively longer period. The improved EPR phenomenon was obtained with e-TSL but not with free DOX. With e-TSL-DOX, induction of increased drug accumulation by improved EPR was evident by 6 hr. These opposing trends in drug accumulation with EPR and preheating between free DOX and e-TSL-DOX is likely because the behavior of a small molecule and a nanoparticle is different in the tissue; tumor perfusion resulting from hyperthermia increases perfusion both in and out of the tissue, and a small molecule like DOX can easily diffuse out of the tumor. And the blood half-life of free DOX in mice (12 min) is significantly shorter than e-TSL (2.03±0.77 hr) [35]. Therefore e-TSL that possesses the long circulating property may have more chances to extravasate into tumor site. In the case of LTSL, the heat treatment was carried out immediately after i.v. injection, and drug accumulation and delayed tumor growth were improved compared to that with local heat exposure after 24 hr. e-TSL with high thermal sensitivity and stability exhibited superior drug accumulation in tumors with increased time (by 6 hr). Similar with the drug accumulation result, the effect of tumor growth delay of e-TSL was better than free DOX–treated groups. Therefore, determination of a treatment schedule is an important factor in yielding improved therapeutic effects with TSLs. High stability under physiological conditions would enable TSLs carrying desired biological factors to stably circulate through blood vessels in a body. Subsequently, external stimulation to the defective site to induce mild hyperthermia could trigger the release of cargo-content in a spatially- and temporally-controlled manner with therapeutically available amounts that assist the self-healing potential of the diseased tissue.

## Conclusion

We have designed and optimized the formulations of highly thermosensitive liposomes by control of DPPC, DSPC, DSPE-PEG, cholesterol, and ELP-lipid conjugates. During the optimization of formulation, we found that the concentration and balance of cholesterol and ELP-lipid conjugates were the main factors in the amount and temperature of drug release. The characteristics of selected formulations were investigated *in vitro* drug release, cryo-TEM analysis, simulation study, DOX accumulation study, and antitumor efficacy study. The results demonstrated that, in our system, e-TSL was very versatile for the modulation of amount and temperature of drug release, as we intended. Our liposome system was confirmed to be highly stable in physiological environments. It is expected that mild hyperthermia before i.v. injection and lag time after i.v. injection would play important role for maximize the drug accumulation. We found that treatment protocol optimization, in addition to the formulation of the liposome, is important for reflecting the advantage of TSL formulation.

## Supporting Information

Figure S1 The bilayer size in xy dimension (top), which equals the bilayer surface areas, and energies (bottom) of simulation systems as functions of time.
(TIF)
