# Supplementary figures and images for: Formulation Optimization and In Vivo Proof-of-Concept Study of Thermosensitive Liposomes Balanced by Phospholipid, Elastin-Like Polypeptide, and Cholesterol
Source: PLoS One. 2014 Jul 28;9(7):e103116. doi: 10.1371/journal.pone.0103116 (PMC4113353; doi:10.1371/journal.pone.0103116)

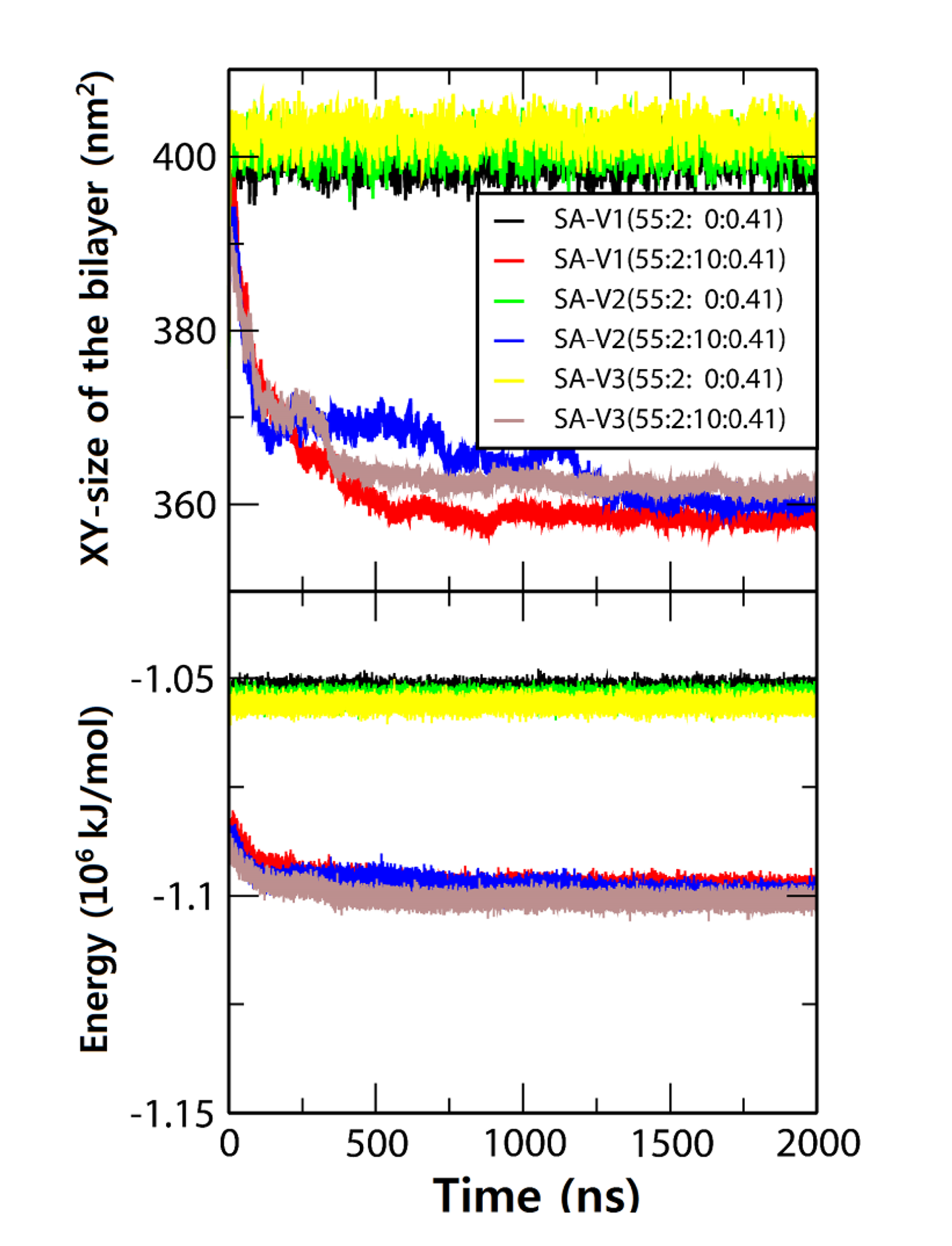

Supplement: Figure S1 — The bilayer size in xy dimension (top), which equals the bilayer surface areas, and energies (bottom) of simulation systems as functions of time. (TIF) [file pone.0103116.s001.tif]
